# Monocytes from infliximab-resistant patients with Crohn’s disease exhibit a disordered cytokine profile

**DOI:** 10.1038/s41598-020-68993-1

**Published:** 2020-07-22

**Authors:** Federica Gaiani, Bianca Maria Rotoli, Francesca Ferrari, Amelia Barilli, Rossana Visigalli, Maria Clotilde Carra, Gian Luigi de’Angelis, Nicola de’Angelis, Valeria Dall’Asta

**Affiliations:** 1 Gastroenterology and Endoscopy Unit, Department of Medicine and Surgery, University of Parma, University Hospital of Parma, Via Gramsci 14, 43126 Parma, Italy; 2 Unit of General Pathology, Department of Medicine and Surgery, University of Parma, Via Volturno 39, 43125 Parma, Italy; 3 Rothschild Hospital, AP-HP, Université de Paris, 5 rue Santerre, 75012 Paris, France; 4 Department of Digestive, Hepatobiliary Surgery and Liver Transplantation, Henri Mondor University Hospital, AP-HP, Université Paris Est-UPEC, 51 avenue du Maréchal de Lattre de Tassigny, 94010 Créteil, France

**Keywords:** Cell biology, Immunology, Gastroenterology

## Abstract

Crohn's disease (CD) is a chronic inflammatory disorder characterized by immune response dysregulation. Tumor necrosis factor-α (TNFα) is a key cytokine in the pathogenesis of CD, as indicated by the efficacy of anti-TNF-α therapy with infliximab (IFX). However, approximately 30–40% of CD patients fail to respond to IFX with still unclear underlying mechanisms. This study compares the inflammatory phenotype of monocytes from CD patients, who respond or non-respond to IFX. Under basal conditions, the mRNA for the cytokines TNFα, IL-23, IL-1β and the chemokines CXCL8/IL-8, CCL5/RANTES and CCL2/MCP-1 was up-regulated in monocytes from non-responders than responders. The expression of the same cytokines and CCL2/MCP-1 was higher in non-responders also upon LPS treatment. Moreover, higher secretion of TNFα, IL-1β, IFNγ and IL-2 proteins occurred in the supernatants of LPS-treated non-responders cells. Resistance to IFX in CD may result from a transcriptional dysregulation of circulating monocytes, leading to hyperactivation of pro-inflammatory pathways. Monocytes’ cytokine profile may thus represent a predictive marker of response to IFX. Monocytes were isolated from blood samples of 19 CD patients (11 responders, 8 non-responders) and incubated with or without LPS. Cytokine profiles were assessed by RT-qPCR and, in the supernatants, by ELISA assay.

## Introduction

Crohn’s disease (CD) is a chronic inflammatory disorder potentially affecting the whole gastrointestinal tract, with segmental and transmural involvement of the gastrointestinal mucosa. Although the precise etiology of the disease is still controversial, there is a general consensus on a multifactorial pathogenesis, including genetic predisposition, environmental factors, and dysregulation of both innate and adaptive immune system responses against intestinal microflora^1^.

The hallmark of CD is a chronic inflammation promoted and sustained by hyperactivated effector immune cells through an increased production of proinflammatory cytokines, mainly tumor necrosis factor (TNF)^2^. Lamina propria macrophages isolated from colonic biopsies of CD patients have been shown to spontaneously produce increased amounts of TNF, that correlate with the degree of tissue involvement and mucosal inflammation^3^, demonstrating their central role in the perpetuation of inflammation.

TNF is synthesized as a membrane-bound protein (mTNF) which is released as soluble form (sTNF) after cleavage of the extracellular domain by TNF-converting enzyme (TACE)^4^. The cytokine is endowed with different functions: by binding to the ubiquitous TNF receptor 1 (TNFR1), sTNF can alternatively activate caspase-dependent apoptosis, and promote proinflammatory cytokine expression through the activation of the transcription factor nuclear factor B (NF-κB)^5^; on the other hand, TNFR2, whose expression is restricted to immune cell system, mainly binds to mTNF to increase lymphocytes T regulators (Tregs) stability, expansion and functions^6^.

The critical relevance of the cytokine in CD is confirmed by the efficacy of anti-TNF antibodies-based therapies, which lead to a clinical response in up to 60% of patients and a long-term maintenance of remission in a large number of subjects (up to 63.45%)^7,8^. Among them, the monoclonal antibody Infliximab (IFX), known as Remicade (Janssen Biotech, Horsham, PA, USA), deserves particular attention, being the first biological response modifier approved by the US Food and Drug Administration (FDA) for the treatment of CD in 1998 and thereby becoming the first inhibitor of TNF employed in the clinical practice^9^. Although the exact mechanisms of action of TNF-targeting agents are still a matter of debate, there is a general consensus that one of their main therapeutic properties is mediated by binding to mTNF that finally leads to the indirect induction of intestinal T cells apoptosis by blocking the TNFR2-dependent signaling^10,11^.

Unfortunately, anti-TNF agents fails to show therapeutic efficacy in a relevant group of CD patients: approximately one-third of patients do not respond to initial anti-TNF therapy (primary nonresponse), while 30–50% of patients lose response along the treatment (secondary nonresponse). The reasons for this failure remain still poorly understood. Among the possible causes, insufficient serum drug levels and the development of neutralizing anti-drug antibodies have been proposed to explain the lack of response^12,13^. Furthermore, the existence of cytokine signaling pathways alternative to TNF has been suggested as a possible cause for therapeutic failure^14^; in this context, a recent study by Schmitt et al. describes an hyperproduction of IL-23 by CD macrophages that is expected to overcome the induction of apoptosis triggered by anti-TNF antibodies, by binding to IL-23R on TNFR2 expressing T cells, causing resistance to the therapy^15^. In this perspective, macrophages should be investigated as major players of resistance mechanisms.

The aim of the present study is to explore the inflammatory phenotype of immune cells isolated from CD patients responding (responders) or not responding (non-responders) to the therapy with IFX, as classified according to endoscopic and clinical criteria, in order to highlight possible differences between the two groups of patients. Since it has been ascertained that the population of mucosal macrophages is constantly replenished by circulating monocytes^16^, the expression of inflammatory markers has been investigated in blood isolated monocytes, so as to evaluate their potential as predictive biomarkers for the response to anti-TNF therapy.

## Results

A total of 19 CD patients (mean age 39.2 years, range 19–78 years) under therapy with IFX were included. Among them, 11 were responders to anti-TNF therapy, and 8 were non-responders. Among non-responders, 2 were primary non-responders, 6 were secondary non-responders. The demographic and clinical features of the study sample are displayed in Table 1. The responder group was comparable to the non-responder group except for the location of CD, with the disease being only ileal for the majority of responders (72.7%) and ileo-colonic for the non-responders (62.5%) (p = 0.043). Only 2 out of 19 patients (10.5%) had comorbidities of hematologic origin; in particular, one patient among the responders presented with essential thrombocythemia, and one patient in the non-responders group presented with polycythemia vera in follow-up. Overall, 3 out of 19 patients (15.8%) were smokers of approximately 1 pack/year, equally distributed between responders and non-responders, 18.2% and 12.5%, respectively.

**Table 1:** Clinical features of the CD patients enrolled.

	Total (n = 19)	Responders (n = 11)	Non-responders (n = 8)	p value
Age [mean (range)]	39.2 (19–78)	40.9 (23–64)	36.9 (19–78)	0.272
**Sex [n (%)]**				
M	13 (68.4)	9 (81.8)	4 (50)	0.319
F	6 (31.6)	2 (18.2)	4 (50)	
BMI [mean (range)]	22.5 (16.6–28.4)	22.5 (16.6–28.4)	22.6 (19–23.6)	1
**Comorbidities [n (%)]**				
None	17 (89.5)	10 (90.9)	7 (87.5)	0.879
Hematologic disorders	2 (10.5)	1 (9.1)	1 (12.5)	
**Ongoing therapy [n (%)]**				
IFX	14 (73.7)	9 (81.8)	5 (62.5)	0.603
IFX + AZA	5 (26.3)	2 (18.2)	3 (37.5)	
**Smoke [n (%)]**				
Yes	3 (15.8)	2 (18.2)	1 (12.5)	1
No	16 (84.2)	9 (81.8)	7 (87.5)	
**CD location (Montreal classification) [n (%)]**				
Ileal	10 (52.6)	8 (72.7)	2 (25)	**0.043**
Colonic	3 (15.8)	2 (18.2)	1 (12.5)	
Ileo-colonic	6 (31.6)	1 (9.1)	5 (62.5)	
**SES-CD [n (%)]**				
Remission	10 (52.6)	10 (90.9)	0 (0)	**< 0.0001**
Mild activity	5 (26.3)	1 (9.1)	4 (50)	
Moderate activity	4 (21.1)	0	4 (50)	
Severe activity	0	0	0	
**CDAI [n (%)]**				
Asymptomatic remission	10 (52.6)	10 (90.9)	0 (0)	**< 0.0001**
Mildly to moderately active	6 (31.6)	1 (9.1)	5 (62.5)	
Moderately to severely active	3 (15.8)	0	3 (37.5)	
Severely active to fulminant	0	0	0	
Serum IFX concentration, µg/ml [mean (range)]	5.25 (1.4–12.9)	5.15 (2.9–7)	5.4 (1.4–12.9)	0.596
**Anti-IFX antibodies [n (%)]**				
Yes	1(5.3)	0	1(12.5)	0.389
No	17 (89.4)	11 (100)	6 (75)	
n/a	1 (5.3)	0	1 (12.5)	

The disease duration varied among patients both in “responders” and in “non-responders”. In particular, the mean duration of the disease was 6.59 years among “responders” (range 0.5–20 years) and 9.06 years among “non-responders” (range 0.5–22 years).

Most of the patients (73.7%) were treated with IFX alone, although some patients (37.5% non-responders vs. 18.2% responders) underwent a combined therapy with IFX and azathioprine, without significant differences between the two groups (p = 0.603). The overall duration of IFX therapy varied among patients in both groups. In particular, the mean duration of IFX therapy was 31 months among “responders” (range 2–87 months) and 26 months among “non-responders” (range 2–70 months).

In accordance with the SES-CD and CDAI indexes, none of the patients presented with severe disease, and, even among non-responders, CD presented with mild to moderate clinical activity in the majority of patients (62.5%). Infliximab serum levels were within the normal ranges in all patients, except for one non-responder, who was treated with the higher dose of IFX (i.e. 10 mg/kg), in accordance with international protocols^17^. Overall, only 1 out of 19 patients had positive anti-IFX antibodies; this patient was not excluded because the IFX serum dose was within the normal range (6.4 µg/ml, normal range 3–7 µg/ml), and the antibody concentration was 10.3 ng/ml, which was at the upper limit of normal (10 ng/ml).

**Figure 1:**
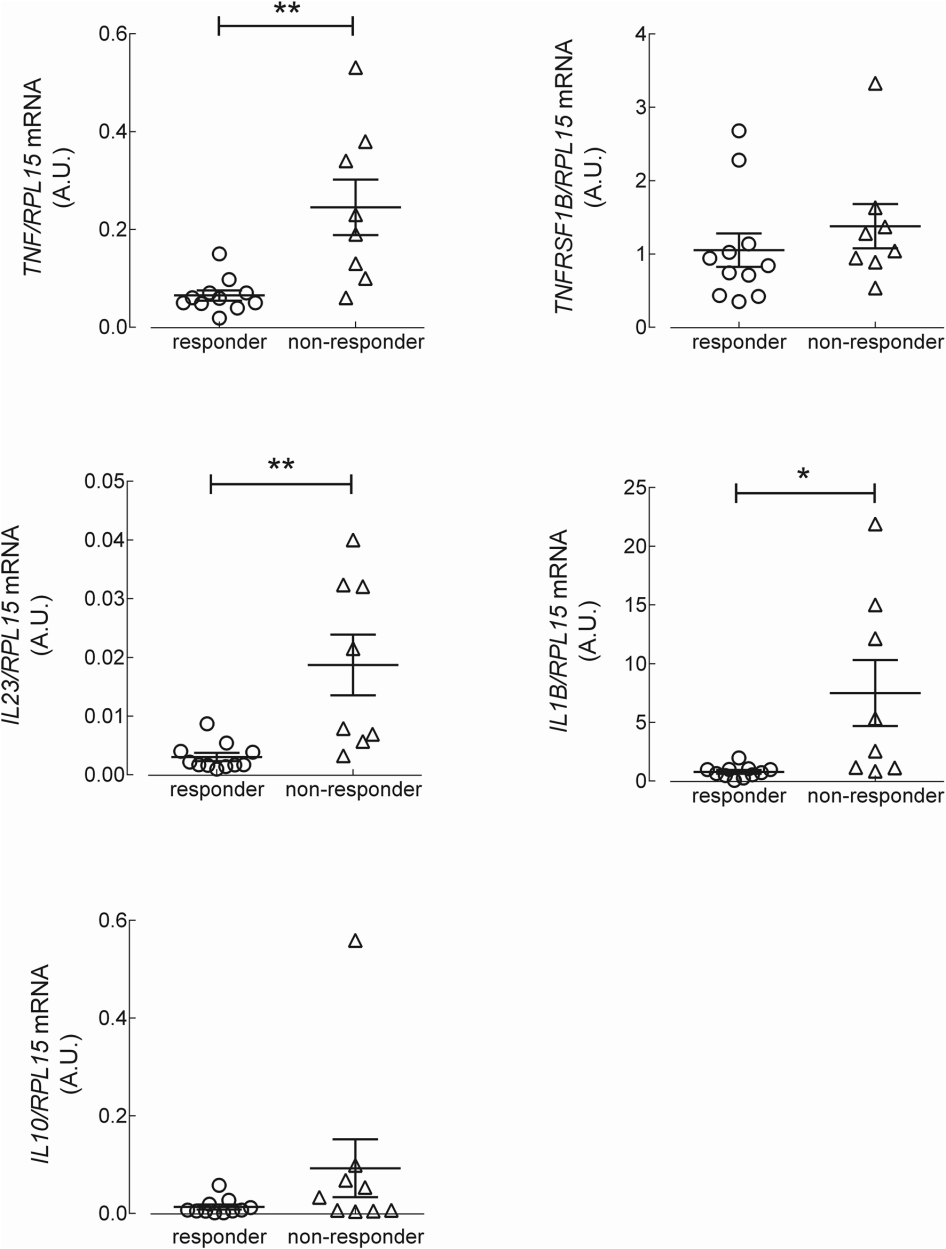
Analysis of gene expression in CD monocytes maintained under basal resting conditions. Cells, isolated from responders (n = 11) and nonresponders (n = 8) to the therapy with IFX, were cultured in complete medium for 4 h; the mRNA for the genes of interest was then analyzed as described in Material and Methods. The amount of the indicated genes, expressed as Arbitrary Unit (A.U.), was normalized for that of the housekeeping gene *RPL15* using the formula 2^ΔCt^ (see “Materials and methods”). Each symbol represents a single patient; the mean ± SEM is indicated as a line. *p < 0.05, **p < 0.01.

**Figure 2:**
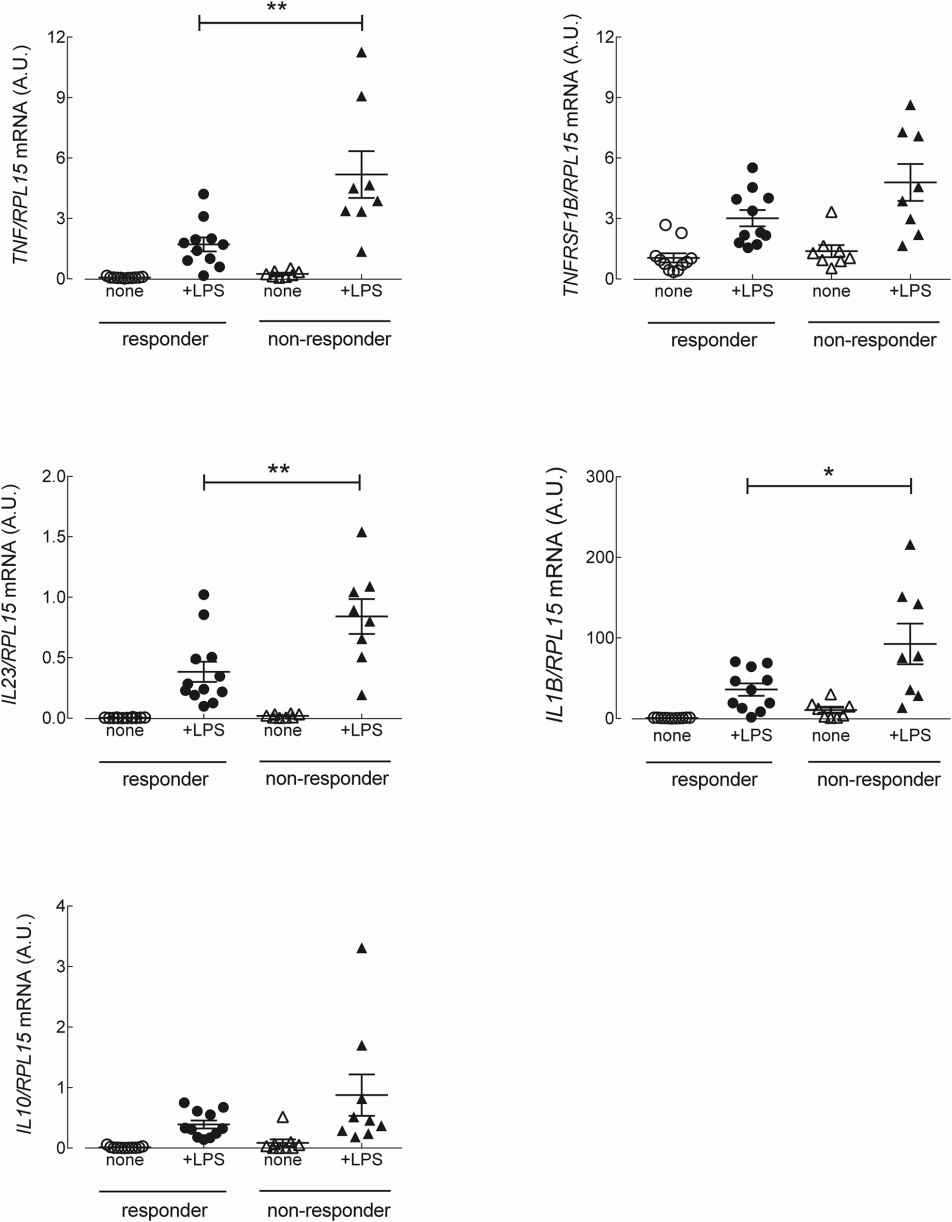
Analysis of gene expression in LPS-stimulated CD monocytes. Cells, isolated as in Figure, were incubated for 4 h either in the absence (none) or in the presence of 0.1 µg/ml lipopolysaccharide (+ LPS). The amount of the indicated genes, expressed as Arbitrary Unit (A.U.), was normalized for that of the housekeeping gene *RPL15* using the formula 2^ΔCt^ (see “Materials and methods”).Each symbol represents a single patient; the mean ± SEM is indicated as a line. *p < 0.05, **p < 0.01.

**Figure 3:**
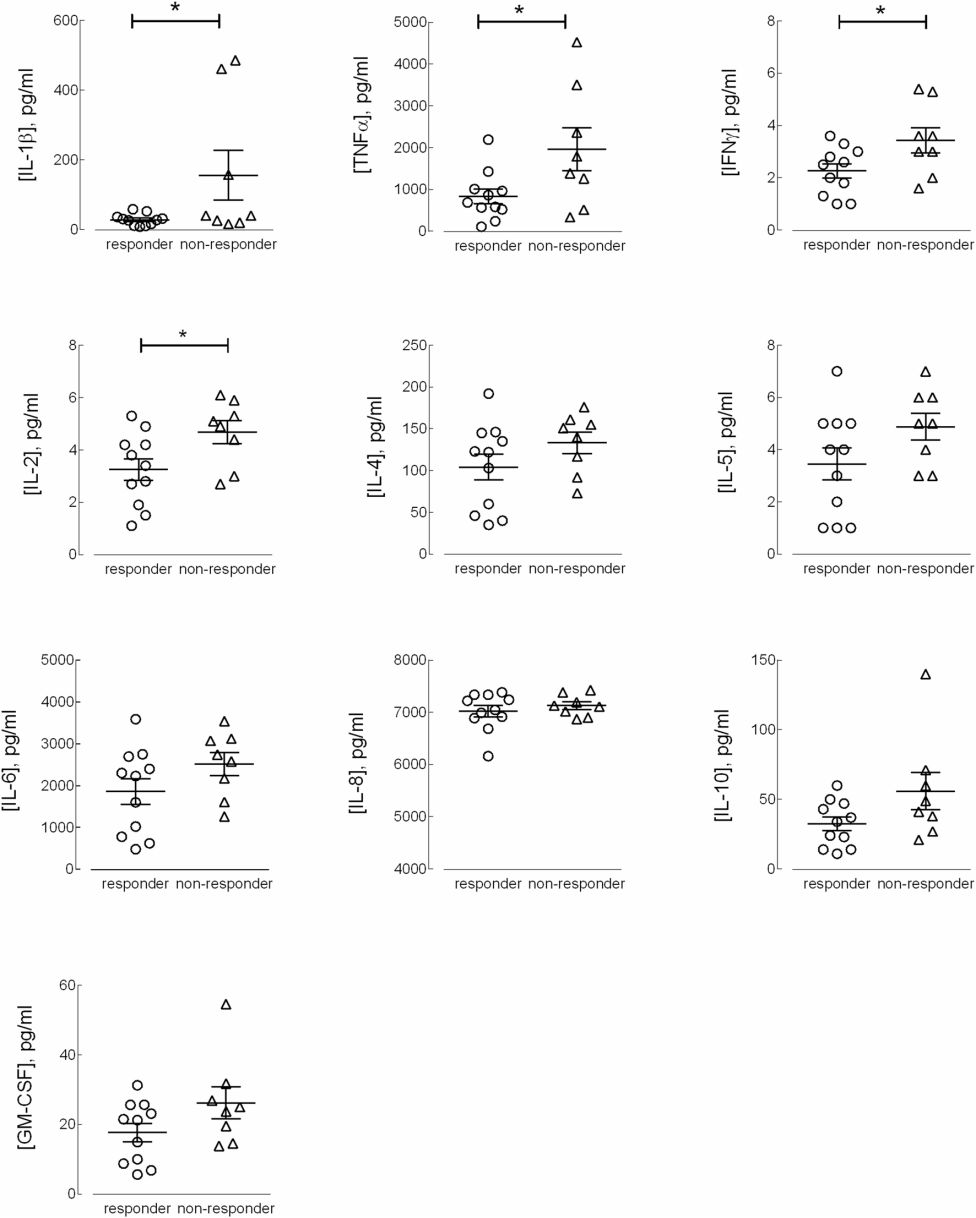
Cytokine secretion by LPS-stimulated CD monocytes. Supernatant from LPS-treated cells as in Figure were assayed for cytokine levels (see “Materials and methods”). Each symbol represents a single patient; the mean ± SEM is indicated as a line. *p < 0.05.

**Figure 4:**
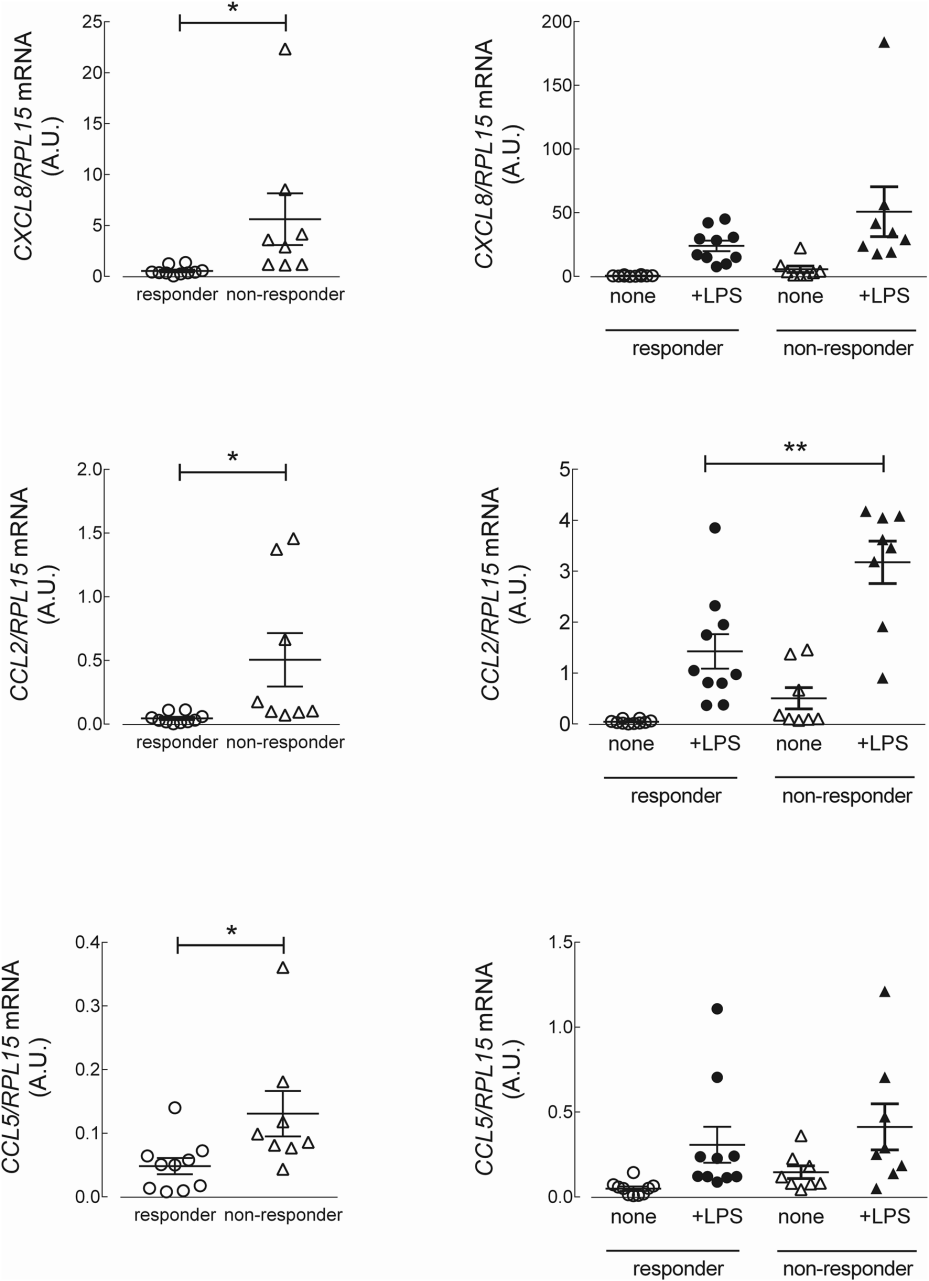
mRNA levels of the chemokines IL-8, MCP-1 and RANTES in CD monocytes maintained under basal conditions or stimulated with LPS. Cells isolated from responders (n = 10) and non-responders (n = 8) to IFX were cultured for 4 h in complete medium, either in the absence (none) or in the presence of 0.1 µg/ml LPS (+ LPS). The amount of the indicated genes, expressed as Arbitrary Unit (A.U.), was normalized for that of the housekeeping gene *RPL15* using the formula 2^ΔCt^ (see “Materials and methods”). Each symbol represents a single patient; the mean ± SEM is indicated as a line. *p < 0.05, **p < 0.01.

To explore the molecular mechanisms responsible for resistance to anti-TNF therapy in CD patients, we next analyzed the expression of inflammatory cytokines in monocytes, both under basal resting conditions and after priming with lipopolysaccharide (LPS). The data presented in Fig. 1 show that under basal resting conditions, the expression level of the mRNAs coding for *TNF*/TNF-α, *IL23*/IL-23 and IL1B/IL-1β were significantly higher in monocytes isolated from CD non-responders than in those from responders. The expression of *TNFRSF1B*/TNFR2 and of the anti-inflammatory cytokine *IL10*/IL-10 did not differ between the two groups of patients. The same analysis, performed on monocytes activated for 4 h with LPS (Fig. 2), demonstrated a marked induction of the tested genes in all CD patients, with *TNF*/TNF-α, *IL23*/IL-23 and IL1B/IL-1β more expressed in non-responders than in responders. Similar results were reached when assaying the supernatants collected from LPS-treated cells for the production of a panel of 10 cytokines (TNF-α, IL-8, IL-1β, IL-6, GM-CSF, IL-10, IL-2, IL-4, IL-5, and IFN-γ). The data obtained (Fig. 3) were consistent with the gene expression data for IL-1β and TNF-α, indicating that these cytokines were secreted at higher levels in non-responders than in responders; a similar pattern was observed for IFNγ and IL-2. The amount of the other cytokines tested (i.e., IL-4, IL-5, IL-6, IL-8, and GM-CSF) was, instead, comparable in the two groups. Finally, the expression of the chemokines *CXCL8*/IL-8, monocyte chemoattractant protein-1 (*CCL2*/MCP1) and *CCL5*/RANTES was tested. As shown in Fig. 4, under basal conditions, all these chemokines were more expressed in monocytes from non-responder than in responders; in LPS-treated cells, only *CCL2* was differentially expressed in the two groups, reaching higher values in cells from non-responder patients.

## Discussion

Despite the widespread use of anti-TNF therapies due to their proven efficacy in patients with CD, the key mechanisms of resistance to TNF-targeting drugs and the perpetuation of mucosal inflammation in CD patients are, thus far, only poorly understood; moreover, there is an unmet clinical need for prognostic tools or predictive biomarkers of resistance to anti-TNF agents^12,14^.

As there is consensus on the crucial role of intestinal macrophages in initiating and continuing inflammation in CD, we here present the results of a prospective study addressing the expression of inflammatory markers in immune cells from CD patients who respond to IFX therapy vs*.* patients who do not.

The clinical characterization of the two groups of patients, responders and non-responders to IFX, highlighted the expected difference in the distribution of SES-CD and CDAI scores. Interestingly, also the location of the disease described by Montreal classification was significantly different between the two groups: ileum was, indeed, involved in 16 out of 19 patients, but among them an isolated ileal localization was more represented among “responders”, while “non-responders” showed an extended ileo-colic disease in 62.5% of cases. The physiopathological mechanism leading to the development of the disease in the small bowel rather than only in the colon is scarcely explored, anyway literature data show that CD involving the small bowel typically manifests more severely, is more difficult to diagnose and to monitor during the follow-up and is more prone to develop complications (fistulas and strictures) than CD limited to the colon^18^.

Once identified the two groups of patients, we next investigated the inflammatory phenotype of blood circulating monocytes, to evaluate their potential as a diagnostic tool for therapy resistance, with an approach similar to that previously employed for celiac disease^19,20^. Results obtained suggest that the failure of therapy with IFX may derive from a deregulation of the cytokine profile of these cells, that associates with the activation of pro-inflammatory pathways. In particular, the expression of TNF-α, IL-1β, IL-23 appears higher in monocytes from non-responder patients than in responders, both under basal conditions and after priming with LPS. Conversely, the difference in expression of the anti-inflammatory cytokine IL-10 did not reach statistical significance. In addition, our study revealed an increased expression of the chemokines IL-8, MCP1 and RANTES in non-responders, with MCP1 differentially expressed in responders and resistant patients also upon LPS treatment. High levels of this chemokine have been described in pathological loci, such as the submucosa and muscularis of Crohn’s disease (CD)^21^, and the MCP1‐A2518G polymorphism is considered a protective factor for inflammatory bowel disease (IBD) in European populations^22^.

In our study, the exclusion of other clinical reasons for IFX failure, i.e. the presence of anti-IFX antibodies or low serum levels of the drug, strengthens the hypothesis of a role for the cytokine profile of circulating monocytes in the development of resistance to anti-TNF agents. For example, in agreement with the study by Schmitt et al., we demonstrate here a significant hyperexpression of IL-23 in innate immune cells, which is supposed to play a key role in the mechanism of resistance to anti-TNF therapy^15^. To this concern, the assessment of a high expression of the cytokine in circulating monocytes could be preventively employed to direct the therapy toward drugs alternative to anti-TNF agents, such as the anti-IL-23 ustekinumab, available in Europe since 2018.

Although this is a single-center prospective study including a limited number of patients, our findings suggest that monocyte analysis could be a reliable tool for the prediction of IFX efficacy; once identified which cytokines can be used as markers of resistance, monocyte typing could be performed in the early phases of induction of therapy, to spare useless and potentially detrimental ineffective infusions for resistant patients and limit the healthcare costs.

## Conclusion

The present study suggests that resistance to IFX therapy in CD patients may result from an innate transcriptional dysregulation of monocytes, which leads to the excessive activation of pro-inflammatory pathways. The definition of a cytokine signature in blood monocytes could, hence, serve as a fingerprint for the early identification of IFX resistant patients, with the aim to optimize the therapeutic approach.

## Materials and methods

### Patient recruitment and monocyte isolation

**Figure 5:**
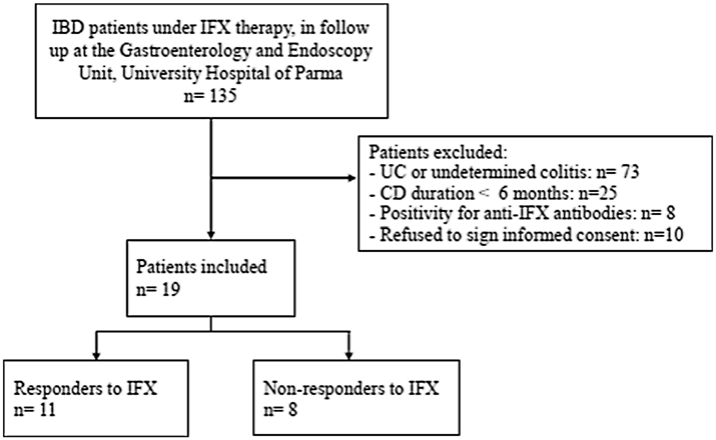
Diagram showing the flow of participants through the study.

CD patients were recruited at the Gastroenterology and Endoscopy Unit of the University Hospital of Parma, Italy, between September 2018 and June 2019 in occasion of their access to the hospital for treatment administration. Adult patients with a histologically confirmed diagnosis of CD of at least 6 months and under therapy with IFX were included in the study. Exclusion criteria included refusal to sign the informed consent form; diagnosis of UC or indetermined colitis; ongoing therapy with corticosteroids; presence of abscess; and contraindications for or reactions to IFX therapy. Demographic and clinical data were extracted from patient records and included body mass index (BMI), comorbidities, and ongoing medical therapies. CD was classified by using the Crohn’s Disease Activity Index (CDAI)^23^ and the Montreal classification for location of the disease^24^ clinically, whereas the Simple Endoscopic Score for Crohn’s Disease (SES-CD) was used to classify the disease endoscopically^25^. All patients underwent endoscopy within 2 months of inclusion in the present study. Patients were then classified as responders to anti-TNF therapy if they had a SES-CD score < 5 and/or CDAI score < 220. A diagram to report the inclusion of participants in the present study is included in Fig. 5. Peripheral blood samples of all patients (approximately 10–12 ml) were taken the day of the scheduled infliximab administration (every 8 weeks), before starting the infusion, and after clinical evaluation. Samples of serum from all the patients were employed for measuring the amount of circulating antibodies against IFX as well as IFX serum concentration by means of an ELISA commercial kit (Lisa Tracker, ALIFAX) according to the manufacturer’s instructions. IFX serum levels were considered in the normal range for values between 3 and 7 µg/ml, and anti-IFX antibodies were considered negative for values < 10 ng/ml^26^. In parallel, circulating monocytes were isolated from blood samples as previously described^27,28^. Briefly, heparinized blood, diluted 1:1 with PBS, was layered on Lympholyte-H gradient medium (EuroCone, Milan, Italy) and centrifuged at 800*g* for 20 min at 20 °C. Peripheral blood mononuclear cells (PBMCs) were collected from the interface and washed in RPMI medium with a centrifugation at 200*g* for 7 min at 20 °C. Cells were then suspended in RPMI containing 10% endotoxin-free fetal bovine serum (FBS) and seeded in 12-well plates. After a 60-min incubation at 37 °C in an atmosphere at 5% CO_2_, nonadherent cells were removed with vigorous washes in prewarmed RPMI medium. Adherent monocytes were cultured for 4 h in RPMI 1640 medium supplemented with 10% FBS, 2 mM l-glutamine, and 1% penicillin/streptomycin in the absence or presence of 0.1 µg/ml lipopolysaccharides from *E. coli* 0111:B4 (LPS). After this period, the culture medium was collected and stored at − 20 °C for the determination of pro-inflammatory cytokines, while cells were employed for the analysis of gene expression upon RNA isolation. The viability of adherent monocytes was assessed by Trypan blue exclusion and was > 98% in all cases; CD14 expression was greater than 90%.

### RT-qPCR analysis

Total RNA was isolated with GeneJET RNA Purification Kit (Thermo Fisher Scientific), according to the manufacturer’s instructions, as previously described^29^. 1 µg of RNA was then reverse transcribed with a RevertAid First Strand cDNA Synthesis Kit (Thermo Fisher Scientific), and qPCR was performed on 20 ng of cDNA by employing a StepOnePlus Real-Time PCR System (Thermo Fisher Scientific). The amount of IL1B (NM_000576.3) and the amount of the reference gene RPL15 (ribosomal protein-like 15, NM_001253379.2) were monitored with specific TaqMan Gene Expression Assays (Thermo Fisher Scientific), while the expression of all other genes was measured using specific forward/reverse primer pairs (Table 2) and SYBR Green PCR Master Mix (Thermo Fisher Scientific). The amount of the genes of interest, expressed as Arbitrary Unit (A.U.), was calculated relative to that of the reference gene using the formula $${2}^{\Delta Ct}$$ (where $$\Delta Ct= {Ct}_{RPL15 }- {Ct}_{gene of interest}$$).

**Table 2:** Sequences of the primer pairs employed for RT-qPCR analysis.

Gene/protein name NCBI Ref Seq identifier	Forward primer	Reverse primer
*CXCL8/*IL8 (NM_000584.4)	ACTGAGAGTGATTGAGAGTGGAC	AACCCTCTGCACCCAGTTTTC
*IL10/*IL-10 (NM_000572.3)	CAAGGCGCATGTGAACTCC	GATGTCAAACTCACTCATGGCT
*IL23/*IL-23 (NM_016584.3)	CTCAGGGACAACAGTCAGTTC	ACAGGGCTATCAGGGAGCA
*CCL2*/MCP-1 NM_002982.4	CAGCCAGATGCAATCAATGCC	TGGAATCCTGAACCCACTTCT
*CCL5/*RANTES (NM_001278736.2)	CCAGCAGTCGTCTTTGTCAC	CTCTGGGTTGGCACACACTT
*TNF/*TNFα (NM_000594.4)	ATGAGCACTGAAAGCATGATCC	GAGGGCTGATTAGAGAGAGGTC
*TNFRSF1B/*TNFR2 (NM_001066.3)	TGAAACATCAGACGTGGTGTG	TGCAAATATCCGTGGATGAAGTC

### Cytokine measurement

Supernatants collected from monocyte cultures were assayed for secreted cytokines with the Bio-Plex System by Bio-Rad by employing the Cytokine Human Magnetic 10-Plex Panel (Thermo Fisher Scientific), which simultaneously measured the levels of TNF-α, IL-8, IL-1β, IL-6, GM-CSF, IL-10, IL-2, IL-4, IL-5, and IFN-γ in each sample. Briefly, all supernatants were clarified prior to the analysis by centrifugation (12,000 × *g* for 10 min at 4 °C); 50 µl of each medium were then added to the plate, previously prepared as required by the manufacturer. After 2 h incubation at room temperature in the dark, the plate was washed twice, then further incubated for 1 h with Biotinylated Detector Antibody. Finally, after 30 min in the presence of Streptavidin-RPE solution, the plate was washed again twice and inserted into the Bio-Plex System by Bio-Rad for the analysis. A standard curve was run in parallel, employing samples provided in the assay; the range of concentration analyzed for each cytokine in the assay employed was (in pg/ml) 6–5,190 for TNF-α, 11–8,150 for IL-8, 12–8,890 for IL-1β, 7–5,120 for IL-6, 7–5,210 for GM-CSF, 12–8,800 for IL-10, 14–10,390 for IL-2, 63–45,800 for IL-4, 10–7,380 for IL-5, and 6–4,450 for IFN-γ. The amount of cytokines in each sample was calculated from the standard curve using the software by Bio-Rad and expressed as pg/ml of incubation medium.

### Materials

Fetal bovine serum and RPMI-1640 medium were purchased from EuroClone (Milano, Italy). Sigma-Aldrich (Milano, Italy) was the source of all other chemicals.

### Statistics

GraphPad Prism 5.0 (GraphPad Software, USA) and SPSS (IBM, Statistics, USA) software were used for the statistical analyses. Clinical data are expressed as frequencies (n, %) or means (standard deviation). Group comparisons were carried out by using the chi-squared test and Mann–Whitney U test for categorical and continuous variables, respectively. Gene expression and cytokine production were analyzed with a two-tailed Student's t-test for unpaired data. Differences were considered statistically significant when p < 0.05.

### Ethical considerations

The study protocol was approved by the local ethical committee (672/2018/OSS/UNIPR approved on 4 September 2018) and conducted in accordance with the principles of the Declaration of Helsinki (1964). Patients were screened for inclusion during follow-up visits for CD therapy administration. All the included patients provided written informed consent before the inclusion in the present study.
